# Severe tetanus following ulcerated skin cancer: Case report

**DOI:** 10.1097/MD.0000000000021529

**Published:** 2020-07-31

**Authors:** Jin Wang, Ying Yang, Chao Yang, Wen Lv, Shihai Xu, Fei Shi, Aijun Shan

**Affiliations:** a Department of Emergency, Shenzhen People's Hospital, The Second Clinical Medical College, Jinan University; The First Affiliated Hospital, Southern University of Science and Technology; b Department of Pediatrics, Futian Women and Children Health Institute, Shenzhen, China.

**Keywords:** tetanus, cancer, propofol, case report

## Abstract

**Rationale:**

Tetanus is usually caused by wound infection with *Clostridium tetani* after acute injuries. Skin cancer wound is a rarely reported cause of *tetani* infection. It is difficult to be diagnosed and mistaken for other brain lesions.

**Patient concerns:**

A 49-year-old man presenting with the only symptom of repeated convulsions was admitted to our department. He had an ulcerated skin cancer on the right buttock that had been excised in another hospital 1 month before admission, leaving the wound unhealed. He was suspected of having a metastatic brain tumor early, but exhibited a negative cranial CT-scan.

**Diagnosis:**

Tetanus was diagnosed when he was observed to have sudden convulsions after sensory stimulation such as noise, light, or touch.

**Interventions:**

Despite administration of a high dose of diazepam and phenobarbitone, continuous generalized rigidity with laryngospasm still occurred. Instead, when propofol was intravenously infused, the spastic convulsion completely stopped. Tracheotomy and mechanical ventilation were performed.

**Outcomes:**

The patient gradually recovered in 2 weeks.

**Lessons:**

Tetanus is rarely infected through the wound of an ulcerated skin cancer. Early diagnosis can only be based on accurate assessment of clinical manifestations, and propofol infusion appears to be more effective in anti-convulsion management for patients with tetanus. Routine vaccination to prevent tetanus in patients with ulcerated skin cancer should be considered in the future clinical work.

## 1 Introduction

Tetanus is a preventable and potentially fatal, muscle-spasm disease caused by *Clostridium tetani*, a motile, spore forming, Gram-positive bacillus.^[1]^ Acute injuries, including needle injuries, lacerations, abrasions, avulsions, frostbite, and burns, are the most frequent causes of *tetani* infection.^[2]^ Furthermore, *Tetani* can also enter human body through chronic wounds, such as pressure ulcer,^[3]^ diabetic complication,^[1]^ and dental cavity.^[4]^ However, cancer has been very rarely reported to result in tetanus.^[5–8]^ Here, we present a rare case of severe tetanus owing to an ulcerated skin cancer on the buttock. With the only symptom of convulsion, the patient was initially suspected of having a metastatic brain lesion from the skin cancer. Tetanus was diagnosed based on typical symptoms that gradually developed. Despite a series of critical problems the patient suffered from, he eventually recovered under the comprehensive and aggressive treatment. This study was approved by the Ethics Committee of Shenzhen People's Hospital. The patient agreed with publication and provided written informed consent.

## 2 Case report

**Figure 1:**
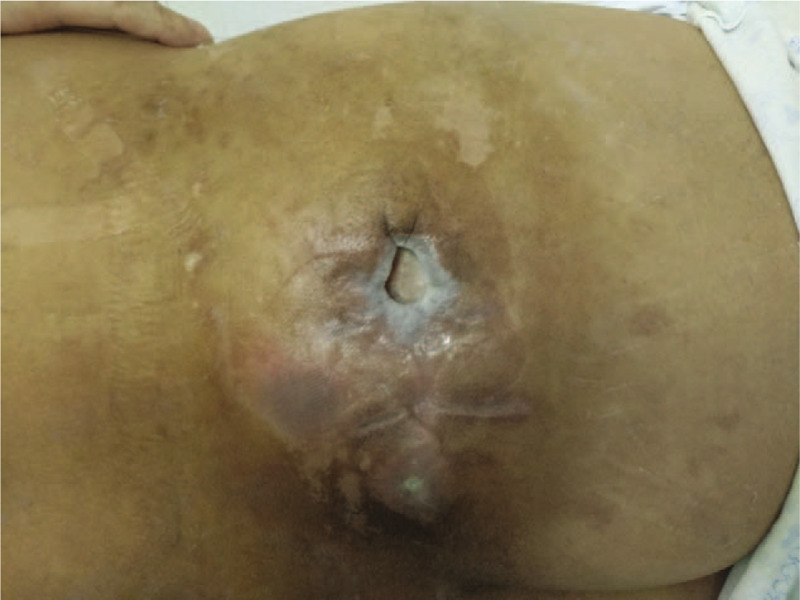
Appearance of the patient's ulcerated skin cancer in the right buttock.

A 49-year-old man presenting with the only symptom of repeated convulsions was admitted to our emergency department. The symptom had been lasting for 5 days (5–6 times per day and 2–5 min every time). He had an ulcerated skin cancer on the right buttock that had been excised in another hospital 1 month before admission. Following excision, subsequent radiotherapy was also administrated, leaving the wound unhealed and a deep ulcer formed (Fig. 1). He had no prior history of trauma/injury or epilepsy.

On admission to department, intracranial metastatic tumor derived from the skin cancer was initially suspected. However, a subsequent cranial computerized tomography (CT) scan showed no metastatic lesion. Cerebrospinal fluid and serum ionised calcium were also normal. Then the patient was transferred to the emergency intensive care unit (EICU) where he was observed to have convulsions after sensory stimulation such as noise, light, or touch. In view of the history of ulcerated skin cancer, the characteristic findings on symptoms and physical examination, we diagnosed tetanus. A dose of 10,000 U human tetanus immunoglobulin (TIG) was injected intramuscularly. Intravenous 10 mg diazepam every 6 h and intramuscular 100 mg phenobarbitone every 8 h were administered to relieve convulsions and spasms. Intravenous 1 g metronidazole every 12 h was administered for anti-infection of *tetani*. Due to a prior history of potential allergy to cephalosporin, penicillin was not utilized for this patient. He had a repeat surgical debridement of the wound.

Thirty-eight hours after admission to EICU he suddenly developed continuous generalised rigidity, opisthotonus (hyperextension of the neck and trunk, flexion of the upper limbs, extension of the lower limbs), and trismus (lockjaw). These symptoms lasting more than 10 min, which could not be reversed by repeated administration of diazepam and phenobarbitone, resulted in fractures of maxillary central incisors. Blood oxygen saturation decreased to 60% and heart rate declined to 40 beats per minute. Given that laryngospasm occurred, tracheal intubation and mechanical ventilation (MV) were implemented under the sedation of continuous intravenous propofol and midazolam. His vital sign rapidly returned back to be normal. The day after MV, a tracheotomy procedure was performed, and the MV was removed.

One week after admission, his rigidity and convulsions were significantly alleviated in severity and frequency, then, he was moved into general ward. He left our department for further oncologic therapy after two more weeks, with the tracheotomy hole sealed. During his hospitalization period in our department, the wound secretion was cultured for three times, while no anaerobic bacteria was detected.

## 3 Discussion

Reports on tetanus caused by that spores entering host through a cancer wound are very rare.^[5–8]^ However, due to timely administration of tetanus antitoxin (TAT) after injury, incidence of acute injury-correlated tetanus has significantly decreased in the last several decades, especially in developed countries. In contrast, with an ageing population and rising incidence of tumor, tetanus caused by chronic wound, including venous leg ulcer, neuropathic diabetic foot ulcer, drug abuse, and ulcerated cancer may be more common in the future.^[1]^ Moreover, chronic wound-related tetanus often affects older individuals that have poorer immunization with more already existing diseases (such as diabetic complications), leading to higher mortality in this subgroup. Two previous studies revealed that it is this subgroup of the population that accounts for 75% of deaths from tetanus.^[9,10]^ Therefore, routine tetanus toxoid vaccination in this high-risk population may be an optimal choice to prevent this severe complication.

An early report containing two cases from China pointed out that the condition of ulcerated cancer-related tetanus was not severe.^[6]^ However, our patient's condition was not optimistic because he had suffered from severe asphyxia, likewise, another severe case was also reported from Japan.^[5]^ So, the mortality risk of cancer-related tetanus should not be underestimated.

In this case, some confounding factors hindered early diagnosis. His primary manifestations were only convulsion and skin cancer, which made us be more concerned about an intracranial metastatic tumor. But sudden exacerbations of convulsions in response to stimuli, and normal CT scan pointed towards a diagnosis of tetanus, further confirmed by the typical symptoms of rigidity, opisthotonus and trismus, as well as the improvement following immunoglobulin treatment. Microbiological culture of wound secretion could not help us find any clues in *Clostridia*. Successful culture was also not available in other reports, reflecting that the diagnosis of tetanus could be largely based on clinical evaluation.^[11]^

Generalized muscular rigidity or spasm is a classical presentation of tetanus. Management of spasm and autonomic instability is the priority in treatment of tetanus. Traditionally, benzodiazepines, including diazepam and phenobarbitone, have been routinely used to control muscle spasms of tetanus.^[12]^ Nevertheless, this patient's severe and continuous generalized stiffness and spasms still occurred despite of application of extreme doses of diazepam and phenobarbitone. In contrast, the administration of propofol revealed excellent anti-spasm effects. Likewise, several reports^[13–15]^ also confirmed the efficacy and safety of propofol in management of tetanus, suggesting that propofol may be a promising choice of pharmacological management of severe tetanus. In addition, the tracheotomy performed for this patient seemed to be redundant, for that the generalized stiffness and laryngospasm were not any more encountered during the afterwards treatment and that MV therapy was removed in the next day. Moreover, the proportion of patients who need tracheotomy also differed greatly among previous studies, varying from 45.6% to 100%.^[16–18]^ Therefore, the prophylactic tracheotomy procedure in management of tetanus should be re-evaluated.

Despite the favorable outcome for tetanus of this patient, the eventual outcome may be not optimistic due to the cancer itself. Also, recurrence of tetanus cannot be absolutely excluded in case of persistent existence of the ulcerated cancer. Routine vaccination to prevent tetanus in patients with ulcerated cancer should be considered in the future clinical work, especially for oncologists.

## Author contributions

**Conceptualization:** Aijun Shan.

**Data curation:** Shihai Xu, Chao Yang and Jin Wang.

**Formal analysis:** Shihai Xu, Chao Yang and Jin Wang.

**Funding acquisition:** Wen Lv, Aijun Shan and Shihai Xu.

**Investigation:** Jin Wang and Ying Yang.

**Methodology:** Jin Wang and Wen Lv.

**Project administration:** Jin Wang, Chao Yang and Fei Shi.

**Resources:** Fei Shi and Aijun Shan.

**Writing – original draft:** Jin Wang and Ying Yang.

**Writing – review & editing:** Aijun Shan, Fei Shi.
